# Impact of High‐Definition Cathodal tDCS Preconditioning on Enhancing the Therapeutic Efficacy of iTBS Combined With FES for Improving Walking Function in Patients With Spinal Cord Injury: A Randomized Controlled Trial

**DOI:** 10.1002/cns.70932

**Published:** 2026-05-16

**Authors:** Huian Chen, Chunya Xia, Weitao Yao, Xuyan Ren, Chengcheng Zhang, Yingjie Fan, Jiaqi Wang, Dejing Cheng, Zelin Su, Huan Du, Yaobo Liu, Min Su

**Affiliations:** ^1^ Department of Rehabilitation Medicine The Fourth Affiliated Hospital of Soochow University (Suzhou Dushu Lake Hospital) Suzhou Jiangsu China; ^2^ Institute of Rehabilitation Soochow University Suzhou Jiangsu China; ^3^ School of Medicine Southeast University Nanjing China; ^4^ Suzhou Ninth People's Hospital Suzhou China; ^5^ Jiangsu Key Laboratory of Drug Discovery and Translational Research for Brain Diseases, Institute of Neuroscience Soochow University Suzhou China; ^6^ Co‐Innovation Center of Neuroregeneration Nantong University Nantong China

**Keywords:** functional electrical stimulation, high‐definition cathodic transcranial direct current stimulation, intermittent theta burst stimulation, spinal cord injury, walking function

## Abstract

**Background:**

Evidence shows functional electrical stimulation (FES) and intermittent theta‐burst stimulation (iTBS) improve motor function in spinal cord injury (SCI). It is unclear whether high‐definition cathodal transcranial direct current stimulation (HD C‐tDCS) preconditioning could enhance the neuromodulatory aftereffect of iTBS to produce greater improvements in walking function.

**Objective:**

This study examined the synergistic effects of combining HD C‐tDCS preconditioning with iTBS and FES therapy on walking ability in individuals with SCI.

**Methods:**

Sixty‐four participants with incomplete SCI were randomly assigned to either active or sham HD C‐tDCS preconditioning, both followed by identical iTBS and FES interventions. Interventions were delivered once daily, five times weekly for four weeks. Both brain stimulation techniques targeted the primary motor cortex (M1) leg area. The primary outcome was the Lower Extremity Motor Score (LEMS). Secondary outcomes included Fugl‐Meyer Assessment of Lower Extremity (FMA‐LE), Berg Balance Scale (BBS), Spinal Cord Independence Measure III (SCIM‐III), Walking Index for Spinal Cord Injury II (WISCI‐II), the root mean square (RMS) values of the rectus femoris and tibialis anterior, step height, knee and ankle range of motion (ROM), and brain functional connectivity via functional near‐infrared spectroscopy (fNIRS), evaluated before and after intervention.

**Results:**

Statistically significant differences were observed in all indices between the two groups before and after intervention (*p* < 0.001). Compared with the control group, the treatment group exhibited a markedly greater improvement in LEMS (MD = 2.12, *p* < 0.001). In addition, the treatment group demonstrated notably more pronounced enhancements than the control group in the FMA‐LE (MD = 1.32, *p* < 0.001), BBS (MD = 3.48, *p* < 0.001), SCIM‐III (MD = 3.27, *p* < 0.001), WISCI‐II (MD = 0.80, *p* < 0.001), RMS values of the rectus femoris (MD = 65.19, *p* < 0.001) and tibialis anterior (MD = 7.20, *p* < 0.001), step height (MD = 44.39, *p* < 0.001), as well as changes in knee (MD = 11.99, *p* < 0.001) and ankle (MD = 8.38, *p* < 0.001) ROM. Furthermore, fNIRS data revealed that the treatment group exhibited significantly stronger functional connectivity across multiple motor‐related cortical networks compared with the control group, including connectivity between the prefrontal cortex (PFC) and M1 (t = 2.92, *p* = 0.021), the right premotor cortex (rPMC) and M1 (t = 4.99, *p* < 0.001), and M1 and primary somatosensory cortex (S1) (t = 4.70, *p* < 0.001).

**Conclusions:**

This study shows that HD C‐tDCS targeting M1 as a preconditioning intervention before iTBS, combined with FES‐assisted walking training, synergistically enhances walking performance and activities of daily living in individuals with SCI. By modulating cortical excitability and promoting motor network reorganization, this sequential multimodal approach enhances neural plasticity and training effects, providing novel mechanistic evidence for mechanistically driven targeted SCI rehabilitation.

## Graphical abstract

This study was divided into two groups: The treatment group, which received HD C‐tDCS combined with iTBS, FES, and conventional therapy, and the control group, which received sham HD C‐tDCS along with identical iTBS, FES, and conventional therapy. Interventions were administered once daily, five days per week, over a four‐week period. Assessments were conducted at two time points: T0 and T1. This study shows that HD C‐tDCS targeting M1 as a preconditioning intervention before iTBS, combined with FES‐assisted walking training, synergistically enhances walking performance and activities of daily living in individuals with SCI. FES, Functional Electrical Stimulation; HD C‐tDCS, High‐Definition Cathodal transcranial Direct Current Stimulation; iTBS, intermittent Theta Burst Stimulation; M1, primary motor cortex; RMT, Resting Motor Threshold.

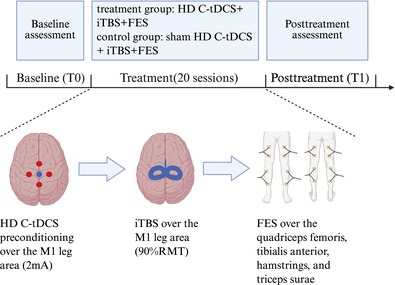

## 1 Introduction

Spinal cord injury (SCI) is a highly disabling central nervous system trauma that impairs sensory, motor, and autonomic function below the injury level to varying degrees [1]. Walking dysfunction, a core issue, severely impacts patients' quality of life and social participation [2]; thus, restoring walking ability is a top priority for maximizing independence [3]. Per guidelines from the International Campaign for Cures of Spinal Cord Injury Paralysis, individuals with American Spinal Cord Injury Association Impairment Scale (AIS) grades C and D have greater potential for high‐level functional recovery than those with grades A and B [4]. For patients with incomplete SCI, the preservation of partial corticospinal tracts and residual neural pathways provides a biological basis for functional recovery through targeted neuroplasticity interventions [5].

Functional electrical stimulation (FES) assisted walking is a promising approach to improve walking function in patients with SCI [6, 7]. By delivering time‐synchronized electrical pulses to target lower limb muscles, FES induces coordinated muscle contractions to complete physiological gait cycles, but the disruption of corticospinal connections after SCI leads to insufficient descending drive from the cerebral motor cortex, which fundamentally limits the long‐term efficacy of FES training [8, 9]. Intermittent theta‐burst stimulation (iTBS), a highly efficient mode of repetitive transcranial magnetic stimulation, can robustly induce long‐term potentiation (LTP)‐like plasticity in the primary motor cortex (M1) and enhance corticospinal excitability to supplement the impaired descending motor drive [10, 11]. The combination of central iTBS and peripheral FES forms a synergistic “central‐peripheral” neuromodulation framework and has been shown to produce greater improvement in walking function than either intervention alone, thereby emerging as a promising treatment strategy for SCI gait rehabilitation [12, 13]. However, chronic SCI triggers maladaptive cortical reorganization, reducing gamma‐aminobutyric acid (GABA)‐mediated inhibitory transmission in M1 and ultimately leading to pathological cortical hyperexcitability [14]. This state significantly reduces the sensitivity, stability, and consistency of M1 neuronal response to iTBS, making it difficult to continuously amplify central descending drive, which is the core bottleneck limiting the efficacy ceiling of the iTBS‐FES combined regimen [15, 16].

To break this bottleneck and optimize the neuromodulatory effect of iTBS, we propose a temporally sequential preconditioning strategy rooted in homeostatic plasticity theory, using high‐definition cathodal transcranial direct current stimulation (HD C‐tDCS) as the preconditioning intervention [17]. Unlike conventional tDCS, which produces diffuse electric fields, HD C‐tDCS delivers a highly focal, precise electric field to the M1 leg region via a multi‐electrode array, enabling targeted modulation of the lower limb motor cortex with minimal off‐target effects [18].

The core rationale of HD C‐tDCS preconditioning lies in homeostatic plasticity theory, which posits that the LTP induction threshold of cortical neurons is dynamically tuned to baseline network excitability [19]. Post‐SCI pathological M1 hyperexcitability markedly raises this threshold, preventing iTBS from inducing stable LTP‐like plasticity—the core mechanism underlying blunted, inconsistent iTBS responses in individuals with SCI [20, 21]. HD C‐tDCS transiently normalizes M1 hyperexcitability by hyperpolarizing the resting membrane potential of cortical neurons [22], restores excitatory‐inhibitory synaptic balance, and lowers the LTP induction threshold. This establishes an optimal M1 “readiness state” that sensitizes neuronal responses to subsequent iTBS, thereby markedly amplifying the neuromodulatory effect of iTBS [23, 24, 25]. Notably, prior exploratory studies have provided preliminary support for this preconditioning strategy. A randomized crossover trial in healthy volunteers demonstrated that HD C‐tDCS preconditioning before iTBS significantly enhanced M1 neural responsiveness, resulting in more than a twofold increase in motor evoked potential (MEP) amplitudes and a marked prolongation of plasticity‐related after‐effects compared with iTBS alone [24]. In addition, this paradigm has also shown promise in post‐stroke upper‐limb motor rehabilitation, where HD C‐tDCS preconditioning plus iTBS yielded significantly greater motor improvements than iTBS alone, providing preliminary clinical support for its application in neurorehabilitation [26].

The innovation of this study is the establishment of a triple neural rehabilitation model featuring a “central preconditioning–central enhancement–peripheral functional closed loop”, which systematically integrates HD C‐tDCS‐mediated cortical state preconditioning, iTBS‐induced M1 excitability enhancement, and FES‐assisted walking training. This model establishes a complete central‐peripheral‐central neuromodulatory loop. While standalone HD C‐tDCS, iTBS, and FES have each shown therapeutic benefits, the efficacy and synergistic mechanisms of HD C‐tDCS‐preconditioned iTBS combined with FES for SCI gait rehabilitation have not been systematically investigated [15].

Accordingly, we conducted a randomized controlled trial (RCT) to test the hypothesis that HD C‐tDCS preconditioning followed by iTBS‐FES combination therapy yields significantly greater gait improvements in individuals with incomplete SCI than iTBS‐FES alone. We further used functional near‐infrared spectroscopy (fNIRS) to assess intervention‐induced changes in motor network functional connectivity to provide auxiliary imaging evidence for the neural mechanisms underlying the regimen. This study will provide high‐level evidence to develop more efficient, mechanism‐driven combinatorial neurorehabilitation strategies for individuals with SCI.

## 2 Materials and Methods

### 2.1 Participants

**FIGURE 1:**
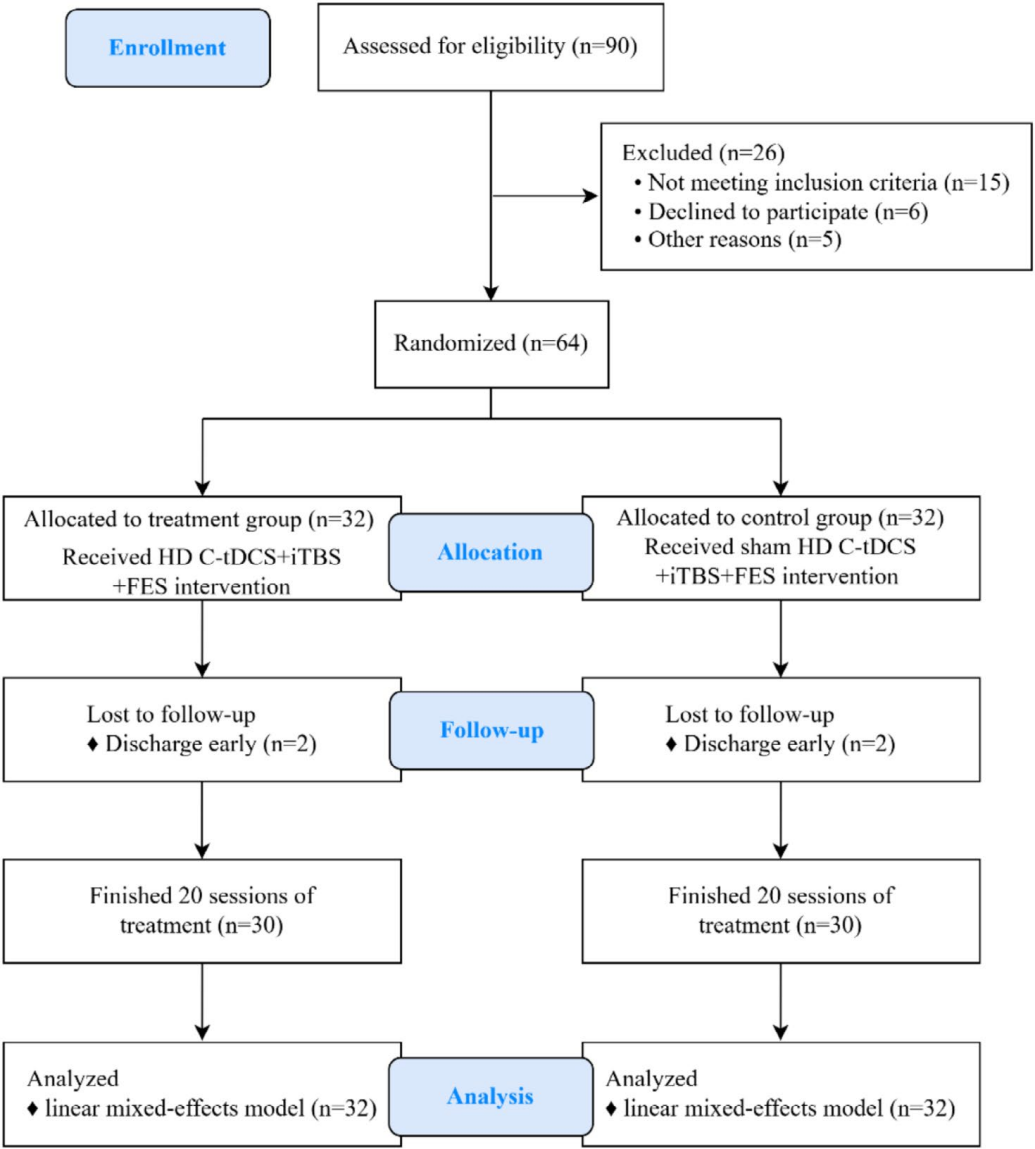
CONSORT diagram of patient flow through the study.

A total of 64 participants with incomplete SCI were recruited from September 2023 to August 2025 in this randomized, sham‐controlled, double‐blind study. All participants were hospitalized in the Department of Rehabilitation, the Fourth Affiliated Hospital of Soochow University. This study has been approved by the Ethics Committee of the Fourth Affiliated Hospital of Soochow University and has been registered in the Chinese Clinical Trial Registry. The CONSORT reporting guidelines for clinical trials were followed in this study (Figure 1).

### 2.2 Inclusion/Exclusion Criteria

The inclusion criteria for participants were as follows: (1) classified as grades C‐D in AIS; (2) the nerve plane is T1‐L2; (3) aged 18–65 years old; (4) stable vital signs and clear consciousness; (5) the course of disease was 1–6 months; (6) informed consent was obtained from the participant or his/her family members.

The exclusion criteria were as follows: (1) history of epilepsy; (2) severe mental illness, severe cognitive and communication impairment, and inability to cooperate with assessment and intervention; (3) wearing a pacemaker, intracranial metal implants, or skull defects; (4) metal implants in the lower limbs; (5) tumor process; (6) severe spasticity, rigidity, contracture, osteoporosis and fractures of the lower limbs.

### 2.3 Randomization and Blinding

Participants were randomly allocated to the treatment group (HD C‐tDCS + iTBS + FES) or control group (sham HD C‐tDCS + iTBS + FES) in a 1:1 ratio via simple randomization. Randomization sequences were generated using the random‐number generator function in Microsoft Excel by study staff uninvolved in recruitment or outcome assessment. The assignment sequences were sealed in sequentially numbered opaque envelopes, opened by research assistants to confirm allocation only after participants completed baseline assessments and confirmed enrollment.

Outcome assessors and participants were blinded. Assessors were unaware of group allocations and took no part in interventions. To maintain participants' blinding, the control group received sham HD C‐tDCS (no actual current output), ensuring participants could not differentiate groups by minimizing somatosensory differences between real and sham stimuli. Research assistants administering the intervention were not blinded but were barred from outcome assessments.

The study period included a baseline assessment period (before the intervention) and an intervention period (consecutive 4 weeks). All outcome measures were measured at 2 time points: Baseline (T0) and after the 4‐week intervention (T1).

### 2.4 Sample Size Calculation

This study was a randomized controlled trial, and the LEMS score was used as the primary outcome index for the improvement of walking function in patients with spinal cord injury. According to the results of the published articles [27], the mean difference and standard deviation of the LEMS score were 8.2 and 10, respectively, with a two‐sided α = 0.05 and a power of 80%. G*Power3.1.9.7 software was used to calculate a sample size of 50 cases. Considering the dropout rate of 20%, the final minimum required total sample size was 64 cases. In the final analysis, a linear mixed‐effects model was used to account for repeated measurements and missing data.

### 2.5 Intervention Protocol

Both groups received identical basic rehabilitation, including ROM, strength, balance, and walking training, for 30 min per day, 5 days per week, over a 4‐week period. In addition, the treatment group underwent HD C‐tDCS preconditioning followed by iTBS and multi‐channel FES‐assisted lower limb walking training, whereas the control group received sham HD C‐tDCS preconditioning followed by the same iTBS and FES protocol. The intervention sequence was standardized as follows: Preconditioning with HD C‐tDCS or sham HD C‐tDCS, followed by iTBS, multi‐channel FES‐assisted walking training of the lower limbs, and concluding with basic rehabilitation training.

### 2.6 HD C‐tDCS

A high‐definition transcranial direct current stimulation device (VC‐8000C; Nanjing Wogao Medical Technology Co. Ltd., China) was used with a 4 × 1 ring electrode configuration (electrode diameter: 12 mm). According to the international 10–20 system, the central cathode was positioned over the leg area of M1, while four return electrodes (C3, C4, Fz, Pz) were arranged concentrically at a distance of 3.5 cm from the target site. The treatment group received a constant current of 2 mA for 20 min, with 30‐s ramp‐up and ramp‐down periods to minimize sensory artifacts. The sham group received the same initial stimulation (2 mA for 30 s) to simulate the onset of cutaneous sensation, followed by no further active stimulation.

### 2.7 iTBS

An NS5000 magnetic stimulator (Wuhan Yiruide Medical Equipment New Technology Co. Ltd., China) was used to deliver iTBS. Using a TMS positioning cap based on the international 10–20 system, an adjustable mechanical arm was employed to fix the center of the conical coil over the Cz point corresponding to the leg area of M1 for stimulation. The stimulation protocol consisted of bursts of three pulses at 50 Hz, repeated at 5 Hz. Stimulation was delivered in 2‐s trains with 8‐s inter‐train intervals, for a total of 600 pulses per session. The stimulation intensity was set at 90% of the resting motor threshold (RMT). In a small number of participants in whom RMT could not be reliably determined, a fixed stimulation intensity of 90% × 80% of the maximal stimulator output (MSO) was used [28]. Surface electromyography (sEMG) was recorded from the affected tibialis anterior muscle, with the recording electrode placed over the muscle belly and the reference electrode positioned on the distal tendon. RMT was defined as the minimum stimulation intensity required to evoke MEPs ≥ 50 μV in at least 5 out of 10 consecutive trials.

### 2.8 Multi‐Channel FES

FES was administered using a biofeedback therapy instrument (RSD RM4; Nanjing Ruishide Medical Technology Co. Ltd., China), with walking training assisted by body‐weight support systems or lower‐limb exoskeleton robots. The target muscles included the quadriceps femoris, tibialis anterior, hamstrings, and triceps surae. Stimulation parameters were set at a frequency of 40 Hz and a pulse width of 250 μs, with alternating activation patterns applied sequentially to the lower limbs to promote coordinated walking. Training duration was 20 min per day.

### 2.9 Primary Outcome

Lower Extremity Motor Scores (LEMS) [29]: Assesses bilateral lower limb muscle strength, including the iliopsoas, quadriceps femoris, tibialis anterior, extensor hallucis longus, and gastrocnemius. Each muscle group is rated on a scale of 0 to 5, with a total possible score ranging from 0 to 50; higher scores reflect better lower limb motor function.

### 2.10 Secondary Outcome

Fugl‐Meyer Assessment‐Lower Extremity (FMA‐LE) was used to evaluate the motor function of lower limbs [30]. The Berg Balance Scale (BBS) was used to evaluate the balance function of SCI patients [31]. The Spinal Cord Independence Measure III (SCIM‐III) was used to evaluate the functional ability of SCI patients [32]. The Walking Index for Spinal Cord Injury II (WISCI‐II) was used to assess the walking ability of patients with SCI [33].

Surface electromyography (sEMG): An SG‐1600B sEMG biofeedback device (Dino Medical Technology Co. Ltd., Zhejiang, China) was used to record the surface electromyographic signals of the rectus femoris and tibialis anterior during walking. Each recording lasted for 3 min, and RMS values were calculated using the middle 1‐min segment to evaluate muscle activation (Figure 3H,I).

Analysis of gait: The Vicon Vero v2.2 motion capture camera (Vicon Motion Systems Ltd) was used to record lower limb kinematics during walking. Reflective markers were placed at the hip joint, 20 cm above the hip joint, knee joint, ankle joint, and toe (Figure 4A–D). 3D visualization of gait analysis was performed in MATLAB using custom scripts.

**FIGURE 2:**
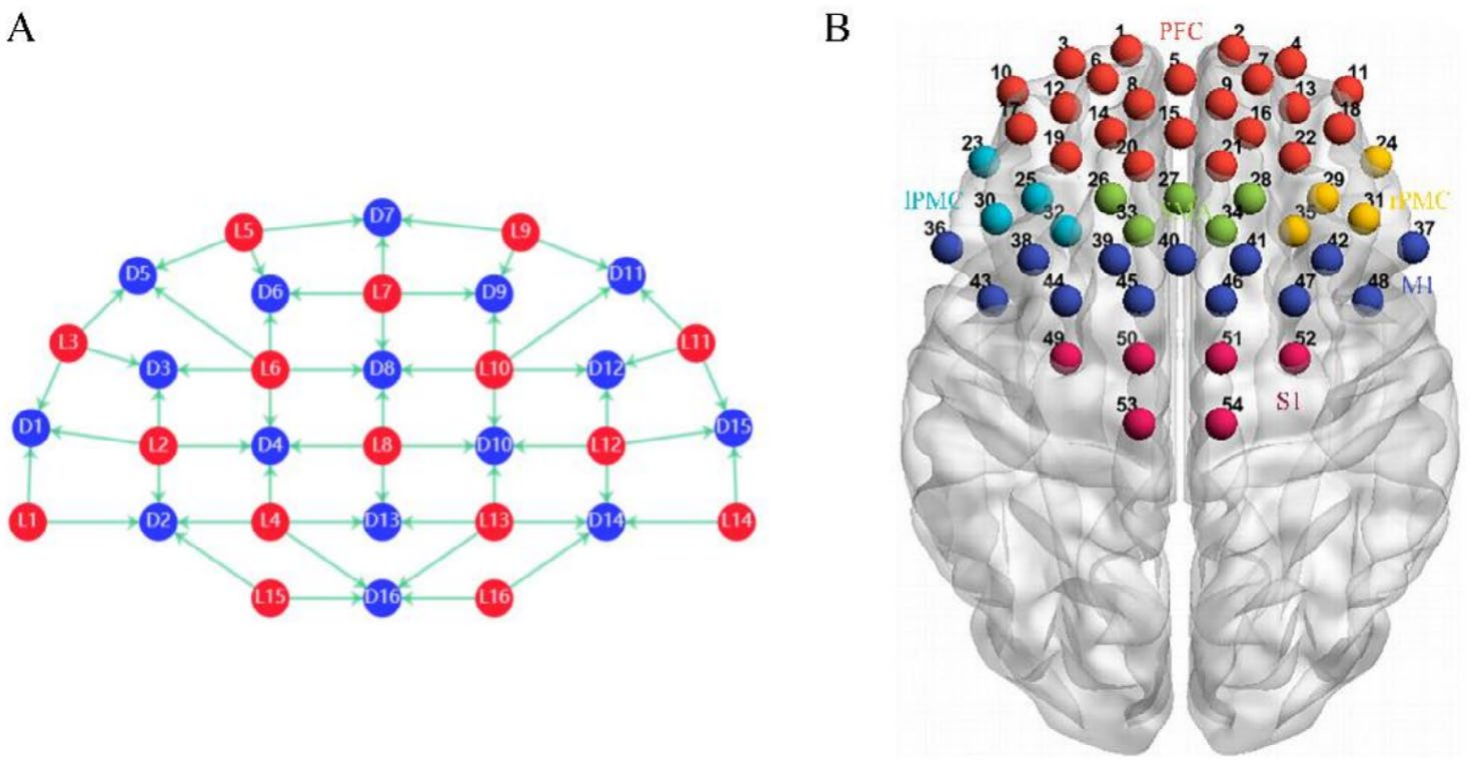
fNIRS probe arrangement and ROI distribution. (A) Source‐detector (SD) arrangement based on the international 10–20 system. (B) Three‐dimensional localization of fNIRS channels and ROIs.

Functional brain imaging system software (C01B‐400S; Suzhou Bairuixin Intelligent Technology Co. Ltd., Jiangsu, China) was used to record the activity of the cerebral cortex in the form of continuous waves of 785 nm and 830 nm to obtain the changes in oxyhemoglobin (HbO), deoxygenated hemoglobin (HbR), and total hemoglobin concentrations. This experiment was based on the internationally used EEG tracing method, the 10–20 system for the localization of brain regions. The optical fiber cap consists of 16 optical emitters and 16 detectors, forming a total of 54 channels (numbered 1–54). (Figure 2A) The spacing was 3 cm, and the sampling period was 0.1 s. Resting‐state data were acquired for 5 min from each patient both before (T0) and after (T1) the intervention. Regions of interest (ROI) included the prefrontal cortex (PFC) (1–22), left premotor cortex (lPMC) (23, 25, 30, 32), supplementary motor area (SMA) (26, 27, 28, 33, 34), right premotor cortex (rPMC) (24, 29, 31, 35), M1 (36–48), and S1 (49–54) (Figure 2B).

fNIRS data were preprocessed and analyzed using dedicated software (Suzhou Bairuixin Intelligent Technology Co. Ltd.). The preprocessing steps were as follows: (1) motion artifacts were identified and corrected using the Temporal Derivative Distribution Repair (TDDR) method; (2) a band‐pass filter (0.01–0.1 Hz) based on a Butterworth filter was applied to the hemodynamic signals to remove high‐frequency noise and low‐frequency drift. (3) The optical density signals were converted into concentration changes of HbO and HbR using the modified Beer–Lambert law. Given its higher sensitivity to cortical activation, HbO was selected for further analysis. The HbO concentration of six ROIs was calculated using an entropy‐weighted averaging method to obtain representative regional signals. Functional brain connectivity was quantified by calculating Pearson correlation coefficients of HbO concentration time series between ROIs during the resting state.

### 2.11 Data Analysis

Statistical analyses were performed using IBM SPSS Statistics 27, and figures were generated with GraphPad Prism 10. Normally distributed data are presented as mean ± SD and compared using independent‐samples *t*‐tests; non‐normally distributed data are expressed as median (IQR) and analyzed with the Wilcoxon rank‐sum test. Categorical variables are reported as *n* (%) and compared using χ^2^ or Fisher's exact test. Linear mixed‐effects models (LMMs) were used to assess changes in LEMS, FMA‐LE, BBS, SCIM‐III, WISCI‐II, RMS of the rectus femoris and tibialis anterior, step height, knee ROM, and ankle ROM. Baseline values were included as covariates; group, time, and group × time interaction were specified as fixed effects, with subject as a random intercept. Models were fitted using restricted maximum likelihood. The primary effect of interest was the group × time interaction. When significant, Bonferroni‐corrected simple effects analyses were performed to examine within‐ and between‐group differences. Missing data were handled within the LMM framework under a missing‐at‐random assumption without imputation.

For fNIRS data, within‐group changes were assessed using paired *t*‐tests and between‐group differences in change scores using independent‐samples *t*‐tests. Multiple comparisons were corrected by the Benjamini–Hochberg procedure to control the false discovery rate (FDR). The NIRS_KIT toolbox was used to generate two‐dimensional *t*‐value maps, with a red–blue diverging color scale representing the magnitude and direction of *t*‐values. Pearson correlation analysis was conducted to evaluate associations between changes in functional connectivity and clinical outcomes. Statistical significance was set at two‐tailed *p* < 0.05.

## 3 Results

A total of 64 participants with SCI were enrolled in this study and randomly assigned in a 1:1 ratio to either the treatment group (*n* = 32) or the control group (*n* = 32). During the study period, 4 participants (2 in each group) discontinued the intervention and outcome assessments owing to loss to follow‐up. Ultimately, 60 participants completed the entire study protocol. In accordance with the intention‐to‐treat principle, all 64 randomized participants were included in the final analysis. Participants lost to follow‐up were not excluded, given that mixed‐effects models are robust to missing data and support valid statistical inference under such circumstances. Statistical analysis showed that there was no significant difference in demographic and clinical characteristics between the two groups (*p* > 0.05) (Table 1). The Intervention protocol was well tolerated, and no significant adverse effects were reported in either group.

**TABLE 1:** Demographic and clinical characteristics of the 2 groups (*n* = 64).

Variable	Treatment	Control	t/χ^2^/z	*p*
Age, years, mean ± SD	44.13 ± 12.18	44.66 ± 13.00	−0.17	0.867^a^
Sex, *n* (%)			1.07	0.302^b^
Male	18 (56.25%)	22 (68.75%)		
Female	14 (43.75%)	10 (31.25%)		
Etiology, *n* (%)			0.33	0.564^b^
Traumatic	23 (71.88%)	25 (78.13%)		
Non‐traumatic	9 (28.12%)	7 (21.87%)		
Plane, *n* (%)			—	0.878^c^
T1−T6	8 (25.00%)	7 (21.88%)		
T7−L1	19 (59.38%)	21 (65.62%)		
L2	5 (15.62%)	4 (12.50%)		
Disease duration, months, median (IQR)	4.00 (2.00)	4.00 (1.00)	−0.42	0.677^d^
AIS, *n* (%)			0.06	0.802^b^
C	16 (50.00%)	15 (46.88%)		
D	16 (50.00%)	17 (53.12%)		
LEMS, mean ± SD	23.31 ± 7.25	23.56 ± 8.01	−0.13	0.896^a^

Abbreviations: AIS, American Spinal Cord Injury Association Impairment Scale; IQR, interquartile range; LEMS, Lower Extremity Motor Scores; SD, standard deviation.

^a^ Two independent‐sample *t*‐test.

^b^ Chi‐squared test.

^c^ Fisher's exact test.

^d^ Mann–Whitney U test.

Linear mixed‐effects models showed significant main effects of time and group across all outcome measures (*p* < 0.001). Given the significant group × time interactions, the following results focus on the interaction effects and Bonferroni‐corrected simple‐effects analyses.

### 3.1 Primary Outcome

**FIGURE 3:**
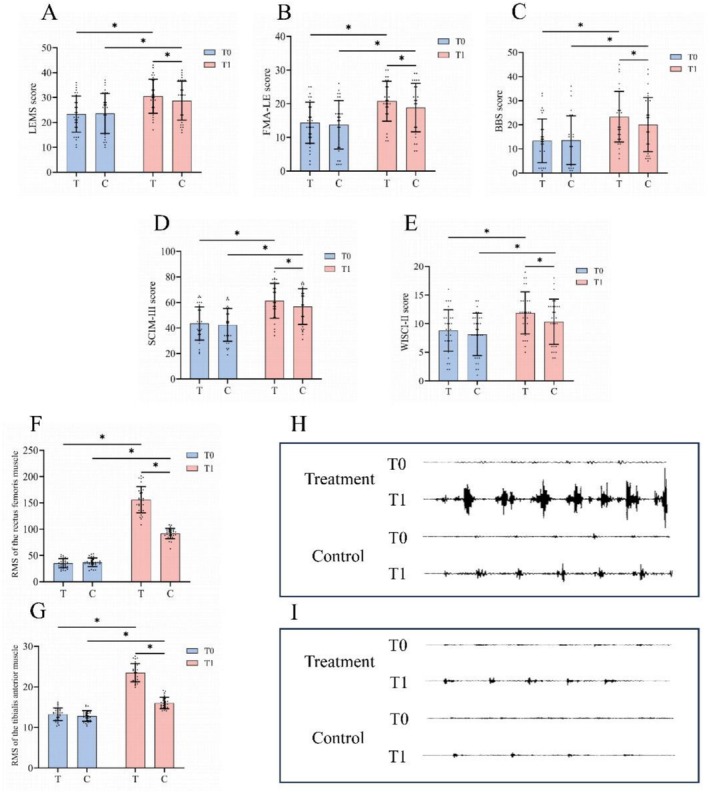
(A–G) Results of Bonferroni‐corrected simple effects analysis for LEMS, FMA‐LE, BBS, SCIM‐III, and WISCI‐II scores, as well as RMS of the rectus femoris and tibialis anterior muscles in the two groups (*n* = 64). (H, I) Representative raw sEMG signals of the rectus femoris (H) and tibialis anterior (I) muscles. T0, before intervention; T1, after intervention; T, treatment group (HD C‐tDCS + iTBS + FES); C, control group (sham HD C‐tDCS + iTBS + FES). LEMS, Lower Extremity Motor Score; FMA‐LE, Fugl‐Meyer Assessment–Lower Extremity; BBS, Berg Balance Scale; SCIM‐III, Spinal Cord Independence Measure III; WISCI‐II, Walking Index for Spinal Cord Injury II; RMS, root mean square. *p < 0.001.

Significant time × group interactions were observed for LEMS (F _(1,63.25)_ = 83.20, *p* < 0.001). No between‐group difference was found at baseline (*p* = 0.951). Both groups improved significantly after treatment (both *p < 0.001*), with greater gains in the treatment group (MD = 7.27, 95% CI: 6.94–7.60) than in the control group (MD = 5.14, 95% CI: 4.81–5.47). Post‐intervention comparison showed a significant between‐group difference favoring the treatment group (MD = 2.12, 95% CI: 1.82–2.42, *p < 0.001*) (Table 2 and Figure 3A).

**TABLE 2:** Bonferroni‐corrected simple effects analyses of outcome measures following significant group × time interactions in linear mixed‐effects models.

Outcome	Statistic	Baseline comparison	Treatment change (T1–T0)	Control change (T1–T0)	Post‐intervention comparison
LEMS	MD, 95% CI	−0.01 (−0.30, 0.28)	7.27 (6.94, 7.60)	5.14 (4.81, 5.47)	2.12 (1.82, 2.42)
	*p*‐value	0.951	< 0.001	< 0.001	< 0.001
FMA‐LE	MD, 95% CI	0.02 (−0.41, 0.45)	6.40 (5.95, 6.85)	5.10 (4.65, 5.55)	1.32 (0.88, 1.76)
	*p*‐value	0.933	< 0.001	< 0.001	< 0.001
BBS	MD, 95% CI	0.01 (−0.62, 0.64)	9.83 (9.15, 10.51)	6.36 (5.68, 7.04)	3.48 (2.82, 4.13)
	*p*‐value	0.977	< 0.001	< 0.001	< 0.001
SCIM‐III	MD, 95% CI	0.01 (−1.32, 1.33)	17.60 (16.25, 18.95)	14.33 (12.98, 15.69)	3.27 (1.91, 4.64)
	*p*‐value	0.993	< 0.001	< 0.001	< 0.001
WISCI‐II	MD, 95% CI	0.00 (−0.23, 0.23)	3.13 (2.90, 3.37)	2.33 (2.10, 2.57)	0.80 (0.56, 1.04)
	*p*‐value	0.995	< 0.001	< 0.001	< 0.001
RMS of the rectus femoris muscle	MD, 95% CI	−0.95 (−7.90, 6.01)	120.77 (113.33, 128.21)	54.63 (47.19, 62.07)	65.19 (58.01, 72.38)
	*p*‐value	0.788	< 0.001	< 0.001	< 0.001
RMS of the tibialis anterior muscle	MD, 95% CI	0.17 (−0.54, 0.87)	10.22 (9.44, 10.99)	3.19 (2.42, 3.96)	7.20 (6.47, 7.92)
	*p*‐value	0.642	< 0.001	< 0.001	< 0.001
Step height	MD, 95% CI	−0.47 (−9.30, 8.37)	97.10 (88.10, 106.11)	52.25 (43.24, 61.26)	44.39 (35.26, 53.52)
	*p*‐value	0.917	< 0.001	< 0.001	< 0.001
Knee joint ROM	MD, 95% CI	−0.20 (−2.47, 2.08)	27.17 (24.78, 29.55)	14.98 (12.59, 17.36)	11.99 (9.64, 14.34)
	*p*‐value	0.863	< 0.001	< 0.001	< 0.001
Ankle joint ROM	MD, 95% CI	0.22 (−0.98, 1.42)	12.70 (11.40, 14.00)	4.54 (3.24, 5.84)	8.38 (7.14, 9.62)
	*p*‐value	0.722	< 0.001	< 0.001	< 0.001

Abbreviations: BBS, Berg Balance Scale; CI, confidence interval; FMA‐LE, Fugl‐Meyer Assessment–Lower Extremity; LEMS, Lower Extremity Motor Score; MD, mean difference; RMS, root mean square; ROM, range of motion; SCIM‐III, Spinal Cord Independence Measure III; T0, before intervention; T1, after intervention; WISCI‐II, Walking Index for Spinal Cord Injury II.

### 3.2 Secondary Outcomes

#### 3.2.1 Lower Limb Motor Function

A significant time × group interaction was identified for FMA‐LE (F _(1,63.04)_ = 16.78, *p < 0.001*), while no difference was observed between groups at baseline (*p* = 0.933). Both groups demonstrated significant post‐treatment improvements (both *p < 0.001*). The magnitude of improvement was greater in the treatment group (MD = 6.40, 95% CI: 5.95–6.85) compared with the control group (MD = 5.10, 95% CI: 4.65–5.55). Accordingly, a significant between‐group difference was observed after intervention (MD = 1.32, 95% CI: 0.88–1.76, *p < 0.001*) (Table 2 and Figure 3B).

#### 3.2.2 Gait and Overall Function

Significant time × group interactions were consistently observed across BBS (F_(1,63.11)_ = 52.23, *p* < 0.001), SCIM‐III (F_(1,62.93)_ = 11.64, *p* < 0.001), and WISCI‐II (F_(1,62.93)_ = 22.74, *p* < 0.001). Baseline comparisons showed no significant differences between groups (all *p* > 0.05). Following treatment, significant improvements were observed in both groups (all *p* < 0.001), with larger gains in the treatment group (BBS: MD = 9.83, 95% CI: 9.15–10.51; SCIM‐III: MD = 17.60, 95% CI: 16.25–18.95; WISCI‐II: MD = 3.13, 95% CI: 2.90–3.37) than in the control group (BBS: MD = 6.36, 95% CI: 5.68–7.04; SCIM‐III: MD = 14.33, 95% CI: 12.98–15.69; WISCI‐II: MD = 2.33, 95% CI: 2.10–2.57). Between‐group comparisons after intervention further confirmed significant advantages for the treatment group (BBS: MD = 3.48, 95% CI: 2.82–4.13; SCIM‐III: MD = 3.27, 95% CI: 1.91–4.64; WISCI‐II: MD = 0.80, 95% CI: 0.56–1.04; all *p* < 0.001) (Table 2 and Figure 3C–E).

#### 3.2.3 Muscle Activity State

Significant time × group interactions were found for the RMS of the rectus femoris (F_(1,63.11)_ = 157.78, *p* < 0.001) and tibialis anterior (F_(1,62.76)_ = 165.79, *p* < 0.001). At baseline, no significant differences were detected between groups (both *p* > 0.05). Both groups exhibited significant increases in muscle activation following treatment (both *p* < 0.001). Notably, the treatment group showed substantially greater improvements (rectus femoris: MD = 120.77, 95% CI: 113.33–128.21; tibialis anterior: MD = 10.22, 95% CI: 9.44–10.99) compared with the control group (rectus femoris: MD = 54.63, 95% CI: 47.19–62.07; tibialis anterior: MD = 3.19, 95% CI: 2.42–3.96). These differences were further supported by significant between‐group effects after intervention (rectus femoris: MD = 65.19, 95% CI: 58.01–72.38; tibialis anterior: MD = 7.20, 95% CI: 6.47–7.92; both *p* < 0.001) (Table 2 and Figure 3F,G).

#### 3.2.4 Gait Biomechanics

**FIGURE 4:**
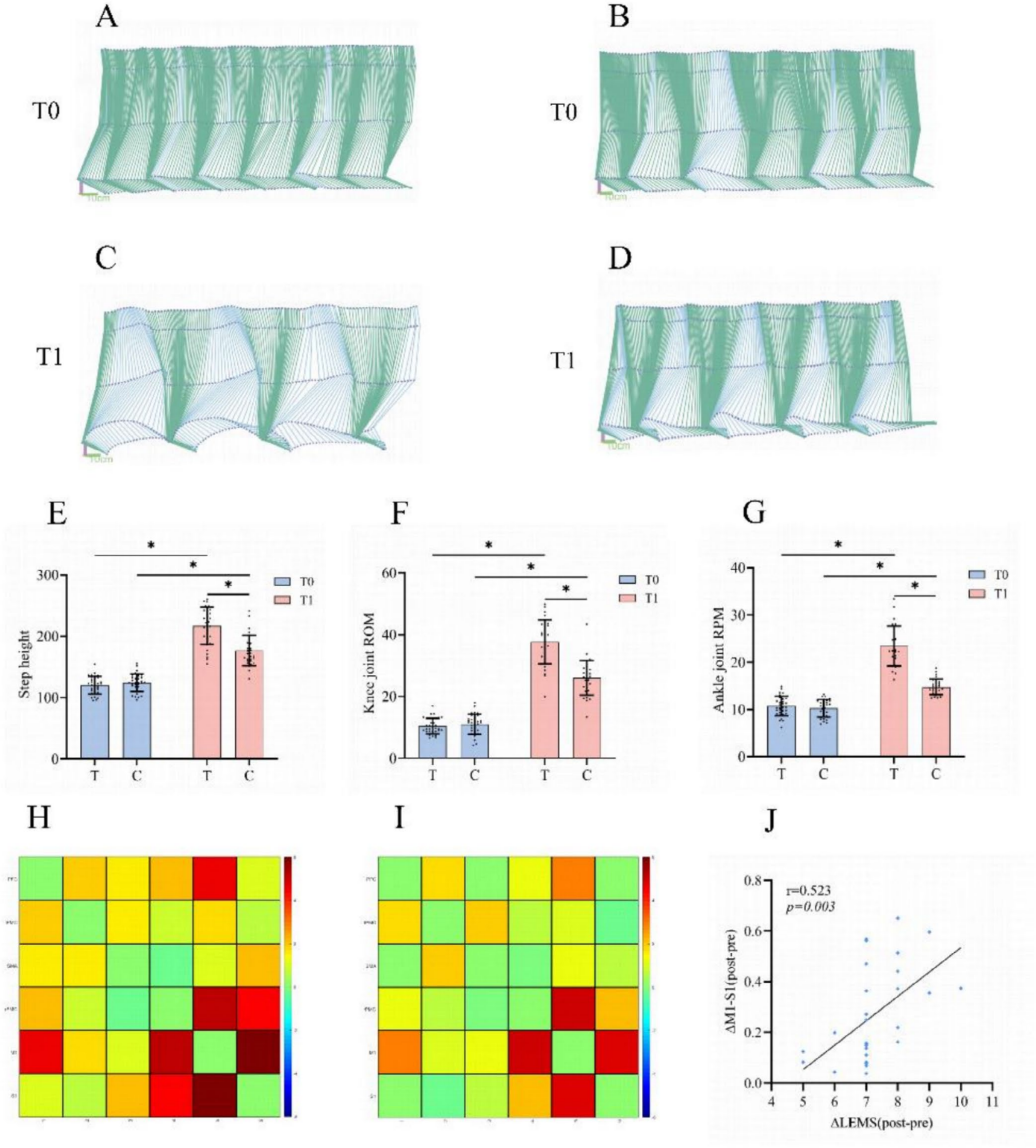
(A, C) 3D gait visualizations of a representative subject in the treatment group at T0 and T1. (B, D) 3D visualizations of a representative subject in the control group at T0 and T1. (E–G) Results of Bonferroni‐corrected simple effects analysis for step height, knee joint ROM, and ankle joint ROM in the two groups (*n* = 64). (H) Within‐group comparison in the treatment group (post vs. pre) showing t‐value maps of functional connectivity. (I) Between‐group comparison based on change scores (Δpost–pre) showing t‐value maps of functional connectivity differences between the two groups. (J) Correlation analysis between changes in LEMS scores and changes in M1–S1 functional connectivity (Δpost–pre) in the treatment group. T0, before intervention; T1, after intervention; T, treatment group (HD C‐tDCS + iTBS + FES); C, control group (sham HD C‐tDCS + iTBS + FES). LEMS, Lower Extremity Motor Score; ROM, range of motion. *p < 0.001.

Significant time × group interactions were observed for all gait kinematic parameters, including step height (F_(1,62.94)_ = 49.54, *p* < 0.001), knee ROM (F_(1,63.02)_ = 52.24, *p* < 0.001), and ankle ROM (F_(1,63.07)_ = 78.39, *p* < 0.001). Baseline values were comparable between groups (all *p* > 0.05). After intervention, both groups demonstrated significant improvements (all *p* < 0.001), with more pronounced changes in the treatment group (step height: MD = 97.10, 95% CI: 88.10–106.11; knee ROM: MD = 27.17, 95% CI: 24.78–29.55; ankle ROM: MD = 12.70, 95% CI: 11.40–14.00) than in the control group (step height: MD = 52.25, 95% CI: 43.24–61.26; knee ROM: MD = 14.98, 95% CI: 12.59–17.36; ankle ROM: MD = 4.54, 95% CI: 3.24–5.84). Consistently, post‐intervention comparisons revealed significant between‐group differences favoring the treatment group (step height: MD = 44.39, 95% CI: 35.26–53.52; knee ROM: MD = 11.99, 95% CI: 9.64–14.34; ankle ROM: MD = 8.38, 95% CI: 7.14–9.62; all *p* < 0.001) (Table 2 and Figure 4E–G).

#### 3.2.5 Brain Functional Connectivity

Two participants in each group were discharged early. Ultimately, fNIRS data were successfully collected from 30 participants in the treatment group and 30 in the control group. No significant changes in resting‐state HbO levels were observed in either group before or after the intervention. Within‐group comparisons (post vs. pre) revealed significant increases in functional connectivity in the treatment group, including PFC–M1 (t = 4.65, *p* < 0.001), rPMC–M1 (t = 5.11, *p* < 0.001), rPMC–S1 (t = 4.48, *p* < 0.001), and M1–S1 (t = 7.69, *p* < 0.001) (Figure 4H and Table S1). No significant changes were observed in the control group. Between‐group comparisons based on change scores (Δpost–pre) demonstrated significant differences in PFC–M1 (t = 2.92, *p* = 0.021), rPMC–M1 (t = 4.99, *p* < 0.001), and M1–S1 (t = 4.70, *p* < 0.001) (Figure 4I and Table S2). Correlation analysis based on change scores further revealed that improvements in LEMS were significantly associated with changes in M1–S1 functional connectivity in the treatment group (*r* = 0.523, *p* = 0.003) (Figure 4J), whereas no significant associations were observed in the control group.

## 4 Discussion

As a highly focused NIBS technique, HD C‐tDCS has demonstrated potential as a preconditioning intervention in the fields of motor function rehabilitation [34], cognitive enhancement [35], and emotional regulation [36] in recent years. Previous studies have shown that HD C‐tDCS can modulate cortical excitability to establish a neurophysiological state more conducive to synaptic plasticity [37], thereby enhancing the effects of subsequent neuromodulation [38]. However, systematic investigations have not yet been reported in the context of SCI rehabilitation, particularly as a preconditioning approach prior to iTBS. This study is the first to apply HD C‐tDCS as a preconditioning strategy in patients with incomplete SCI, aiming to evaluate its synergistic effect on improving walking function when combined with an iTBS and FES program.

The results demonstrated that the treatment group, which underwent HD C‐tDCS preconditioning prior to iTBS combined with FES, exhibited significantly greater improvements than the control group in lower extremity motor function (LEMS, FMA‐LE), balance, walking ability, and activities of daily living independence (BBS, WISCI‐II, SCIM‐III). In addition, enhanced muscle activation (RMS of the rectus femoris and tibialis anterior), improved gait biomechanics (step height, knee, and ankle range of motion), and increased brain functional connectivity were observed. These findings support our central hypothesis that modulating cortical excitability through preconditioning can potentiate subsequent neuromodulatory interventions and facilitate functional recovery.

The primary mechanism underlying the preconditioning effect is the normalization of pathological cortical hyperexcitability within the M1 leg area, which resets the threshold for LTP induction and increases neuronal responsiveness to subsequent iTBS. HD C‐tDCS implemented using a focal 4 × 1 ring electrode configuration enables targeted modulation of the M1 leg representation by inducing a mild hyperpolarization of the resting membrane potential in cortical pyramidal neurons while minimizing current spread to adjacent regions [39, 40]. This transient hyperpolarization contributes to the restoration of excitatory–inhibitory synaptic balance, a critical step in re‐establishing the set point of motor cortical plasticity [34]. Evidence from healthy individuals indicates that HD C‐tDCS preconditioning lowers the LTP induction threshold for iTBS and prolongs the duration of plastic after‐effects beyond 30 min [24], which is consistent with our findings of enhanced lower limb muscle activation and improved gait performance. In patients with SCI, normalization of M1 excitability represents not merely a passive reduction of hyperexcitability but an active recalibration of cortical network responsiveness to neuromodulation. In the absence of such preconditioning, iTBS targeting M1 and the primary somatosensory cortex (S1) has been shown to fail in inducing LTP‐like plasticity in SCI populations, resulting in attenuated and variable improvements in descending motor drive, which represents a limitation of prior iTBS monotherapy studies [41]. Therefore, HD C‐tDCS preconditioning effectively addresses this constraint, enabling iTBS to more fully enhance corticospinal excitability and promote functional recovery in SCI rehabilitation.

Specifically, HD C‐tDCS preconditioning modulates the responsiveness of cortical circuits, enabling subsequent iTBS to more effectively increase corticospinal excitability, elevate MEP amplitudes, and strengthen descending drive from M1 to spinal motor neurons [42, 43]. Peripheral FES then delivers temporally synchronized stimulation to lower limb muscles (e.g., quadriceps and tibialis anterior), eliciting coordinated contractions that mimic physiological gait patterns and generate afferent sensorimotor feedback [44]. This feedback is conveyed to S1 and M1 via ascending pathways, reinforcing LTP‐like plasticity induced by iTBS and forming a positive feedback loop linking central plasticity, peripheral execution, and subsequent consolidation [45, 46]. Compared with conventional iTBS‐FES paradigms, which lack a preconditioning phase, this closed‐loop design may more effectively couple enhanced cortical excitability with peripheral motor output. Consistent with this mechanism, our fNIRS findings demonstrated significantly increased functional connectivity within key motor‐related networks (M1–S1, rPMC–M1, and PFC–M1) in the treatment group, whereas no comparable changes were observed in the control group. Notably, M1–S1 connectivity was positively correlated with improvements in LEMS scores, suggesting that strengthened sensorimotor integration is associated with lower limb functional recovery. These network‐level adaptations indicate that HD C‐tDCS preconditioning does not act in isolation but rather facilitates system‐level reorganization, thereby amplifying the synergistic effects of central iTBS and peripheral FES within a closed‐loop neuromodulation framework.

Preconditioning strategies using HD C‐tDCS have been increasingly explored as a means to enhance the efficacy of non‐invasive brain stimulation. In stroke populations, HD C‐tDCS applied over the affected M1 prior to iTBS has been associated with greater improvements in upper limb motor function (Fugl‐Meyer Assessment‐Upper Extremity, Motricity Index‐Upper Extremity, and modified Barthel Index) compared with iTBS alone [26]. Similarly, studies in healthy individuals have shown that cathodal tDCS preconditioning can prolong corticospinal excitability following rTMS [34] and augment functional connectivity within prefrontal–cingulate networks after iTBS [36]. Together, these findings suggest that preconditioning may enhance the responsiveness of cortical networks to subsequent stimulation. Consistent with this literature, our results extend these observations to the SCI population and support the potential value of HD C‐tDCS‐based preconditioning for improving lower limb rehabilitation outcomes. However, treatment effects may not be uniform across patients with SCI, given the heterogeneity in neurological severity, injury level, etiology, disease duration, and residual neural pathway integrity. Importantly, as our study included only individuals with incomplete SCI (AIS C‐D), the present findings are applicable only to this subgroup. Further studies are needed to identify suitable candidates, optimize stimulation parameters, confirm long‐term efficacy, and support clinical implementation.

This study has several limitations. First, long‐term follow‐up was not conducted because of time and resource constraints. Second, sEMG and 3D gait data were not collected synchronously, which limited temporal alignment analyses and precluded a more detailed investigation into the relationship between muscle activation and gait improvement. Third, resting‐state fNIRS may be more susceptible to variability; therefore, future studies should further validate the neural mechanisms observed in the present study by integrating task‐based fNIRS with functional magnetic resonance imaging (fMRI) or electroencephalography (EEG). Future research should address these limitations.

## 5 Conclusion

In conclusion, this study demonstrates that preconditioning with HD C‐tDCS targeting the leg area of M1 prior to iTBS combined with FES significantly improves walking function and activities of daily living in individuals with SCI. The protocol validates the practical feasibility of precisely timed, closed‐loop rehabilitation by promoting functional reorganization of the entire motor network through multi‐level synergistic interventions, thereby achieving additive therapeutic effects. Mechanistically, HD C‐tDCS likely modulates cortical excitability to enhance the efficacy of iTBS‐induced neuroplasticity; when integrated with FES‐assisted walking training, this combination strengthens motor cortical functional connectivity and overall neural network function. This work provides robust preliminary evidence and novel insights for the development of more effective, mechanism‐driven combinatorial strategies in SCI neurological rehabilitation.

## Author Contributions

Conceptualization: M.S., H.D., and Y.L. Formal analysis: M.S., H.C., and X.R. Funding acquisition: M.S. Investigation: H.C., C.X., W.Y., J.W., D.C., and C.Z. Methodology: M.S., H.C., and Y.L. Project administration: M.S. Resources: M.S. Supervision: H.D., and Y.L. Writing – original draft: H.C., C.X., Y.F., and Z.S. Writing – review and editing: M.S., H.D., and Y.L.

## Funding

This work was supported by the Qilu Medical Research Fund Project of Soochow Medical College of Soochow University (No. 24QL200219) and the National Natural Science Foundation of China (82272594).

## Ethics Statement

This study has been approved by the Ethics Committee of the Fourth Affiliated Hospital of Soochow University (ID: 20230082). The study protocol has been registered in the Chinese Clinical Trial Registry (ChiCTR2300074528) and has strictly followed the requirements of the Declaration of Helsinki. Written informed consent was obtained from all participants or their legal guardians before enrollment.

## Conflicts of Interest

The authors declare no conflicts of interest.

## Supporting information


**Table S1:** Within‐group comparison of functional connectivity in the treatment group before and after intervention.
**Table S2:** Between‐group comparison of changes in functional connectivity between the treatment and control groups.

**Data S2:** CONSORT 2025 checklist.

## Data Availability

The data that support the findings of this study are available on request from the corresponding author. The data are not publicly available due to privacy or ethical restrictions.
