# Supplementary material for: Impact of High‐Definition Cathodal tDCS Preconditioning on Enhancing the Therapeutic Efficacy of iTBS Combined With FES for Improving Walking Function in Patients With Spinal Cord Injury: A Randomized Controlled Trial
Source: CNS Neurosci Ther. 2026 May 16;32(5):e70932. doi: 10.1002/cns.70932 (PMC13179583; doi:10.1002/cns.70932)
Supplement: Supplementary file 1 — Table S1: Within‐group comparison of functional connectivity in the treatment group before and after intervention. Table S2: Between‐group comparison of changes in functional connectivity between the treatment and control groups. [file CNS-32-e70932-s002.docx]

Table S1. Within-group comparison of functional connectivity in the treatment group before and after intervention.

| Brain functional connectivity | Before intervention, Mean ± SD | After intervention, Mean ± SD | t | *p*-value |
| --- | --- | --- | --- | --- |
| PFC–M1 | 0.45±0.20 | 0.62±0.25 | 4.65 | ＜0.001 |
| rPMC–M1 | 0.44±0.24 | 0.65±0.22 | 5.11 | ＜0.001 |
| rPMC–S1 | 0.46±0.25 | 0.65±0.23 | 4.48 | ＜0.001 |
| M1–S1 | 0.36±0.24 | 0.64±0.26 | 7.69 | ＜0.001 |

Abbreviations: SD, standard deviation; PFC, prefrontal cortex; M1, primary motor cortex; rPMC, right premotor cortex; S1, primary somatosensory cortex.

Table S2. Between-group comparison of changes in functional connectivity between the treatment and control groups.

| Brain functional connectivity | Treatment group, Mean ± SD | Control group, Mean ± SD | *t* | *p*-value |
| --- | --- | --- | --- | --- |
| PFC–M1 | 0.17±0.20 | 0.02±0.21 | 2.92 | 0.021 |
| rPMC–M1 | 0.22±0.23 | -0.01±0.07 | 4.99 | ＜0.001 |
| M1–S1 | 0.27±0.19 | 0.02±0.22 | 4.70 | ＜0.001 |

Abbreviations: SD, standard deviation; PFC, prefrontal cortex; M1, primary motor cortex; rPMC, right premotor cortex; S1, primary somatosensory cortex.

**Analysis method**

Near-infrared brain functional imaging system software (Suzhou Bairuixin Intelligent Technology Co., Ltd.) was used for data preprocessing and analysis. Firstly, Temporal Derivative Distribution Repair (TDDR) is used to identify and remove motion artifacts, and the collected blood oxygen signal is processed by 0.01-0.1Hz bandpass filter based on Butterworth filter. Eliminate the interference of high-frequency noise and slow drift. The optical density signals were converted into concentration changes of HbO and HbR using the modified Beer–Lambert law. In view of the high sensitivity of oxygenated hemoglobin data, this study conducted further analysis based on oxygenated hemoglobin data to calculate the average HbO2 concentration and brain network connectivity coefficient in 6 regions of interest (PFC, lPMC, SMA, rPMC, M1, S1).

(1) The HbO2 concentration in the Region of Interest (ROI) of the baseline state and the task state is calculated based on the entropy weight average method, and then the mean value is calculated based on the blood oxygen concentration in the ROI.

The specific calculation process of the entropy weight average method is as follows: Assume that the region of interest YROI contains N channels, and each channel y has K sampling points. First, we max-min all the sampled points in each channel y:

$$y_{ij}^{'}= \frac{y_{ij}- {min(y_{tj})}_{t=1,2,..,K}}{{max(y_{tj})}_{t=1,2,..,K}- {min(y_{tj})}_{t=1,2,..,K}}$$

Where i=1,2, …K,j=1,2, … N,x_ij_∈X.

Then, the probability value p_ij_ of each channel sampling point in the ROI is calculated:

$$p_{ij}= \frac{y_{ij}^{'}}{\sum_{t=1}^{M} y_{tj}^{'}}$$

Where i=1, 2, ... K, j=1, 2, ... N.

Secondly, the information entropy e_j_ of each channel in the ROI is calculated, and the numerical range is within [0,1]:

$$e_{j}= -\frac{1}{ln(n)}\sum_{i=1}^{n} p_{ij}ln(p_{ij})$$

According to the information entropy e_j_, the weight w_j_ of each channel in the ROI is calculated:

$$w_{j}= \frac{1- e_{j}}{\sum(1- e_{j})}$$

Finally, according to the weight w_j_ of each channel, the blood oxygen concentration signal Y_ROI_ of the ROI brain region is calculated:

$$Y_{ROI}= \sum_{j} x_{ij}w_{j}$$

(2) Calculate the mean value according to the blood oxygen concentration data of the resting-state, and the calculation process is as follows:

$$Mean=\frac{1}{n}\sum_{i=1}^{n} x_{i}$$

Where i=1,2, ... n, n is the number of sample points.

(3) Calculate the correlation coefficient between brain regions according to the blood oxygen concentration data of the ROI brain region of the resting-state, and the calculation process is as follows:

Firstly, the Pearson correlation coefficient between rois was calculated, and the adjacency matrix of 6×6 was constructed. The Pearson correlation coefficient was calculated by the following formula:

$$r = \frac{\sum_{i=1}^{n} (r_{i}-\bar{r})(u_{i}-\bar{u})}{\sqrt{\sum_{i=1}^{n} {(r_{i}-\bar{r})}^{2}\sum_{i=1}^{n} {(u_{i}-\bar{u})}^{2}}}$$

Here, r represents the Pearson correlation coefficient between the r brain region and the u brain region, and n is the number of sample points. r_i_ indicates the i-th sample point of the r-th brain region, and u_i_ indicates the i-th sample point of the u-th brain region. r̄ represents the mean of all sample points in the r-th brain region, and ū represents the mean of all sample points in the u-th brain region.
